# How Our Continuing Studies of the Pre-clinical Inbred Mouse Models of Mesothelioma Have Influenced the Development of New Therapies

**DOI:** 10.3389/fphar.2022.858557

**Published:** 2022-03-31

**Authors:** Bruce W.S. Robinson, Alec J. Redwood, Jenette Creaney

**Affiliations:** ^1^ Medicine, University of Western Australia, Perth, WA, Australia; ^2^ Institute for Respiratory Health, University of Western Australia, Perth, WA, Australia; ^3^ Biomedical Science, University of Western Australia, Perth, WA, Australia

**Keywords:** immunothearpy, mesothelioma, chemotherapy, asbestos, cancer

## Abstract

Asbestos-induced preclinical mouse models of mesothelioma produce tumors that are very similar to those that develop in humans and thus represent an ideal platform to study this rare, universally fatal tumor type. Our team and a number of other research groups have established such models as a stepping stone to new treatments, including chemotherapy, immunotherapy and other approaches that have been/are being translated into clinical trials. In some cases this work has led to changes in mesothelioma treatment practice and over the last 30 years these models and studies have led to trials which have improved the response rate in mesothelioma from less than 10% to over 50%. Mouse models have had a vital role in that improvement and will continue to play a key role in the future success of mesothelioma immunotherapy. In this review we focus only on these original inbred mouse models, the large number of preclinical studies conducted using them and their contribution to current and future clinical therapy for mesothelioma.

## 1 Introduction

Mouse models of mesothelioma continue to inform the development of novel treatments for this cancer. In parallel with our human mesothelioma studies, we established a mouse model of the disease that could be used to study and test new treatments. At that time there were no effective therapies for mesothelioma so, realizing that novel therapeutic approaches would be required we set about developing an animal model to test them, and thus we began our preclinical program. Previous studies had established that asbestos caused mesothelioma in rats and mice (Wagner and Berry, 1969) however there were no models of mesothelioma available in inbred mouse strains, a necessary foundation for therapy studies, especially immunotherapy. So starting from scratch we showed that intraperitoneal injection of crocidolite asbestos into mice produced mesothelioma tumors that were very similar to those that develop in humans. We then generated cell lines from multiple tumors that arose in three strains of mice, BALB/c, CBA and C57Bl/6 (Davis et al., 1992). These lines have been used in studies of the biology and immunology of mesothelioma and have provided the platform for testing a variety of new therapies, including chemotherapy, immunotherapy, gene therapy and others. Some of our pre-clinical studies have been successfully translated into the clinic, and all of this work provides insights into the host-tumor interaction. Mouse mesothelioma models represent one of the few sets of animal models of human cancer in which the tumor parallels its human counterpart in terms of carcinogen, tissue of origin, clinical behavior and biology (Davis et al., 1992). In this review, we outline our research journey and role from a time when mesothelioma patients were only offered palliative treatment to today where a number of treatments generate clinical responses, and thus highlight the translational implications of work in this mouse model.

## 2 Chemotherapy and Mesothelioma

### 2.1 Murine Studies

Before 1990 there was no effective, tolerated chemotherapy for mesothelioma. Some agents, such as anthracyclines, were used in selected patients but the response rates were so low and the side effects so disabling that many centers, including our own, opted solely for palliation for mesothelioma patients (Krarup-Hansen and Hansen, 1991). Indeed, there was substantial pessimism in the field of oncology that any chemotherapy would be effective in mesothelioma (Lee et al., 2002); it seemed to be a totally resistant cancer. With that in mind we used the murine mesothelioma model to test the efficacy of a number of, as yet, untrialled chemotherapies including the gemcitabine-cisplatin combination that was being used at that time in lung cancer (Nowak et al., 2002a; Nowak et al., 2003a), as well as cyclophosphamide (van der Most et al., 2009) and, over the years, a variety of other chemotherapeutic agents (Aston et al., 2017).

**FIGURE 1:**
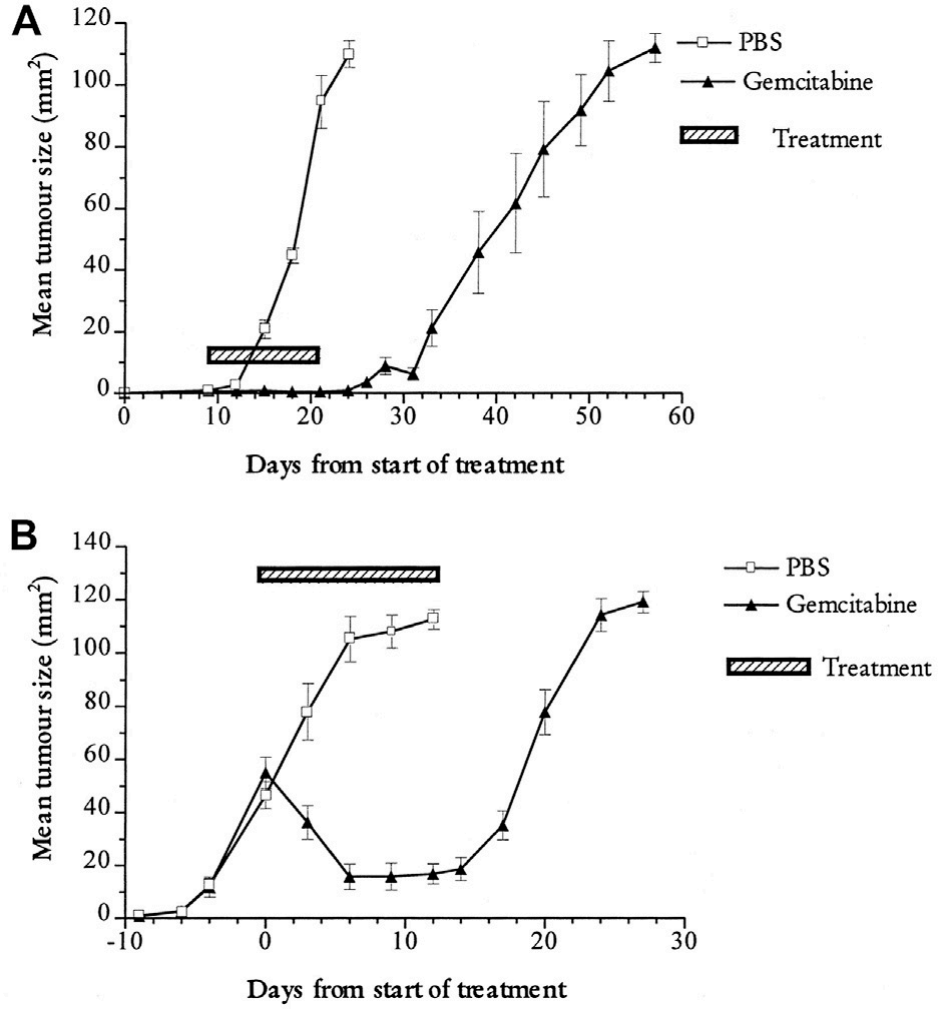
Preclinical evidence of effectiveness of chemotherapy in mesothelioma. The apoptosis-inducing chemotherapy agent gemcitabine (black triangles) induces *in vivo* regression of the mouse mesothelioma tumour line AB1-HA compared to mice treated with phosphate buffered saline alone (open squares) **(A)** Early treatment; tumour bearing mice were treated when tumours were small (∼10 mm^2^) **(B)** Late treatment. tumour bearing mice were treated when tumours were small (∼10 mm^2^). Mice were treated with five doses of gemcitabine or PBS injection at third daily intervals. Figure originally published in Nowak et al., 2002a. Cancer Research 62:2353-8.

In our early study we showed that gemcitabine-cisplatin combination could successfully reduce the growth of both small and large mesothelioma tumors growing in mice (Nowak et al., 2002a) (Figure 1). These encouraging results led us to test these agents in a series of clinical trials.

### 2.2 Clinical Trials and Translation

In the original single centre study of 21 patients, an objective partial response rate of 48% was seen with symptom improvement in 57% of patients receiving intravenous cisplatin and gemcitabine (Byrne et al., 1999). A subsequent multicenter phase II study showed a partial response rate of 33% and stable disease in 60%, with a median survival of 17.3 months (Nowak et al., 2002b). This combination was established as standard clinical care practice (Nowak et al., 2004), one of the first chemotherapies for this disease to produce such good response rates. Not only did data generated in the mouse model lead to a change in clinical practice, they revealed previously poorly understood effects of chemotherapy on the immune system that continue to underpin current novel chemo-immunotherapy trials (Nowak et al., 2002a).

## 3 The Immunobiology of Mesothelioma and the Development of Immunotherapy

The prevailing wisdom about cancer and immunity when we first began these preclinical studies in mesothelioma was that the host immune system was “ignorant” of the presence of cancers, so tumors were able to grow without hindrance from the immune system. However, there has long been clinical evidence that mesothelioma is a potentially immunogenic tumor (Robinson et al., 2001). Inbred strains allowed for immune studies so we utilized these mouse models to demonstrate that mesothelioma is immunogenic, showing that the tumor can be recognized by the immune system and destroyed (Manning et al., 1993). In a program of immunobiological studies of murine mesothelioma (Davis et al., 1993; Bielefeldt-Ohmann et al., 1994) we characterized the inflammatory/immune environment (Robinson et al., 1993), including the involved inflammatory cytokines (Bielefeldt-Ohmann et al., 1994; Fitzpatrick et al., 1995). We, further showed the role regulatory T cells played in controlling the anti-mesothelioma immune response (Gibson et al., 1989; Davis et al., 1992).

### 3.1 Evidence for Immune Engagement

In order to understand if and how mesothelioma tumors engaged with the host immune system, something that was unknown at the time, we needed to create ways of tracking specific anti-mesothelioma immunity. As there were no mesothelioma antigens known at that time, we created mesothelioma cell lines that carried a stably transfected “nominal” antigen (a viral antigen that could be the target of immune attack target), and combined this with the adoptive transfer of T-cell receptor transgenic lymphocytes with specificity for this “neoantigen” (the equivalent to a T-cell monoclonal antibody) to track and explore if, and where, mesothelioma antigens were presented to the immune system (Marzo et al., 1999a).

**FIGURE 2:**
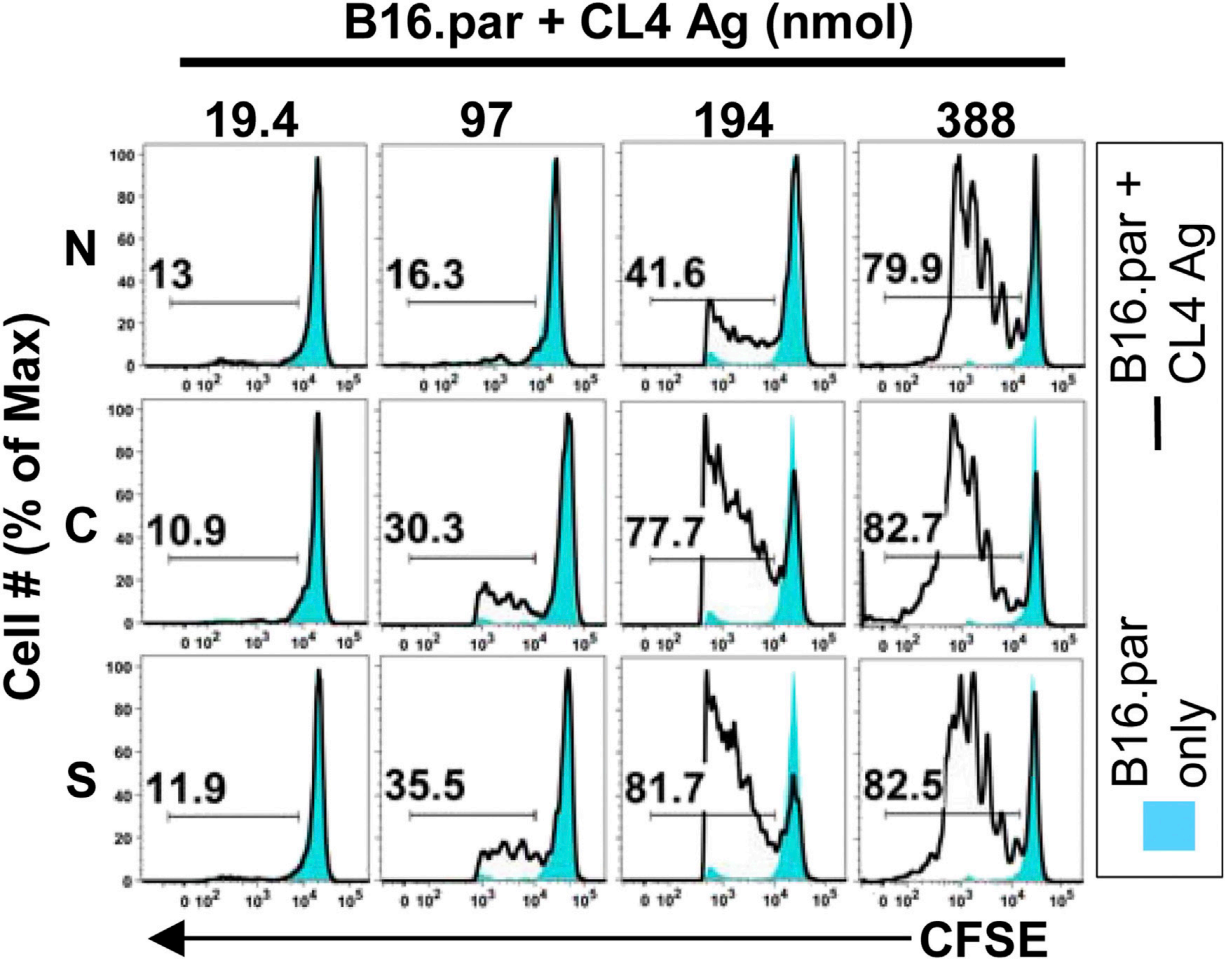
Cross presentation is sensitive to low antigen loads. Cross presentation was assessed using the *in vivo* Lyons-Parish assay (Lyons and Parish, 1994), in which carboxyfluorescein succinimidyl ester (CFSE) stained neoantigen specific T cells (CL4) were intravenously injected into F1 mice bearing the melanoma tumour (B16. par) bearing totals of 19.4, 97, 194 or 388 nmol of the neoantigen CL4 in either the nucleus (N), cytoplasm (C) or supernatant (S). Proliferation of T cells in the tumor draining lymph node on day 11 is shown by the number and size of peaks to the left of the parental non-proliferating single blue peak. Figure originally published in Anyaegbu et al., 2014. PLOS One 9: e107894.

We initially turned our attention to the lymph nodes that drain the tumor, where tumor-specific T cells are educated and activated. Somewhat to our surprise, mesothelioma antigens readily reached these tumor draining lymph nodes (dLN) and were presented to the immune system there (a process known as “cross presentation”) (Marzo et al., 1999b). This process proved to be highly efficient for all antigens tested (Kurts et al., 2010), occurred within 1–2 weeks of the commencement of tumor growth (Marzo et al., 1999a; Marzo et al., 1999b; Robinson et al., 1999) and was highly sensitive, requiring only 200 nmol of tumor neoantigen within the tumor mass to be “visible” to the host immune system (Anyaegbu et al., 2014) (Figure 2). These were the first studies to disprove the hypothesis that tumors grew in hosts because the immune system was ignorant of their presence. These studies also helped us to understand the role of CD4 help in the “post-licensing” of mesothelioma-specific CD8 T cells, a process that is critical in helping CD8 T cells enter the tumor and induce rejection (Marzo et al., 2000).

We then turned our attention to the tumor itself, where tumor-specific T cells need to enter tumors then attack and destroy the tumor cells (McDonnell et al., 2010). We showed that dendritic cells within the tumor, which may be required to restimulate T cells as they enter this site, were, in contrast, to those in tumor dLN, completely unable to present mesothelioma neoantigens to T cells (McDonnell et al., 2015a). Importantly, chemotherapy that destroys tumor cells delivers antigens to the dendritic cells within the tumor and reverses their inability to cross present neoantigen. This enhanced neoantigen presentation within the tumor allows for T-cell restimulation and enhanced anti-tumor effects (Kurts et al., 2010; McDonnell et al., 2015b). These and other ongoing studies of the relationship between mesothelioma and the immune system continue to drive us forward to test novel therapies for mesothelioma, and it has underpinned many of the novel clinical trials described below.

### 3.2 Harnessing the Immune Response to Control Tumor Growth

Having shown that the host immune system is not ignorant of the tumor, we began to test different immunotherapeutic strategies to bolster the anti-mesothelioma immune response with the goal of developing new treatment approaches (Robinson et al., 1993). These original immunotherapeutic strategies involved the administration to mesothelioma-bearing animals of systemic cytokines, such as recombinant interferon alpha (IFNα) (Bielefeldt-Ohmann et al., 1995) and also activating anti-CD40 antibodies (Stumbles et al., 2004; Khong et al., 2012). The latter agent being shown to ligate CD40 and activate dendritic cells. These studies confirmed that mesothelioma could be effectively controlled if the immune system was appropriately engaged, at least by the cytokine IFNα and by anti-CD40, which induced cures of established mesothelioma tumors in the majority of mice studied (van der Most et al., 2005).

#### 3.2.1 Clinical Trials and Translation

These pre-clinical studies underpinned many of the early immunotherapy clinical trials in mesothelioma. Recombinant IFNα was administered subcutaneously, and to our surprise, some spectacular tumor regressions were seen (Christmas et al., 1993). However, although these responses were generally associated with prolonged relapse-free survival lasting some years, they were seen in only a minority of patients (12%) with the remainder of the patients suffering the disabling flu-like illness typically associated with this type of therapy, and we did not consider it ethical to continue it in our patients. It did however confirm that immunotherapy could be effective in patients with mesothelioma and this knowledge has driven our team forward to continue to examine other immunotherapies for mesothelioma.

### 3.3 Manipulating the Immune Response With Gene Therapy

One of the many attractive features of this mouse model is the capacity to genetically manipulate the tumor cells prior to their injection, in order to dissect the anti-mesothelioma immune response. We began by transfecting mesothelioma cell lines to express foreign transplantation (“allo”) antigens to make the mesothelioma cells look like a foreign organ transplant and be rejected, in the process stimulating a broader immune response against the untransfected parental tumor. This proved especially useful in mesothelioma lines that were otherwise ‘non-immunogenic’ (Leong et al., 1994). The expression of allogeneic MHC molecules in a highly immunosuppressive, non-immunogenic murine malignant mesothelioma cell line did led to tumor rejection, however this did not generate systemic immunity sufficient to protect from the parental cell line (Leong et al., 1994).

Transfecting these non-immunogenic mesothelioma cell lines with the immune co-stimulatory molecules B7.1 (CD80) but not B7.2 (CD86) induced and maintained a T-cell response to these mesothelioma tumors, but the response required ongoing expression of B7-1 and the upregulation of major histocompatibility complex class II expression and did not protect against the parental cell line (Leong et al., 1995; Leong et al., 1996; Marzo et al., 1997). Transfection of cytokines such as interleukin-2 (Leong et al., 1997) generated *in vivo* immunity to the mesothelioma tumor that was relatively weak and/or subject to down-regulation so that consistent rejection of unmodified parental tumor cells was not achieved. Transfection of both of the interleukin-12 genes generated a higher level of immunogencity which could control tumor growth (Caminschi et al., 1998). We showed that when cytokine genes were delivered using a vaccinia virus rather than gene transfection the growth of advanced mesothelioma tumors could also be controlled but not eradicated (Upham et al., 1995). These studies enabled us to gain an improved understanding of how a host responds, or fails to respond, to mesothelioma (Robinson et al., 1998; Nowak et al., 2002c; Nelson et al., 2005; van der Most et al., 2006a).

#### 3.3.1 Clinical Trials and Translation

Based upon these preclinical studies that showed that cytokines within a mesothelioma tumor could induce regression, we conducted an immunogene therapy trial in patients with mesothelioma. We used a modified vaccinia virus in which the interleukin-2 gene was inserted into the thymidine kinase region of the virus and the construct was then injected directly into the tumor providing a source of interleukin-2 inside the tumor without viral replication in normal cells (Mukherjee et al., 2000). We found this therapy to be safe and allowed persistent transgene expression in the tumor over 3 weeks, regardless of anti-vaccinia IgG levels, however the amount of interleukin-2 produced in the tumors was insufficient to justify further clinical trials. The study did show that the vector itself would be safe for the delivery of other molecules in such studies. Gene therapy using IFNα has been well studied (Sterman et al., 2011).

### 3.4 Loco-regional Immunotherapy

We have tried a number of loco-regional approaches to mesothelioma immunotherapy using these inbred mouse mesothelioma models. Because the immunosuppressive molecule TGF-β was known to be produced by mouse, as well as human mesothelioma tumor cells, we tested the efficacy of locally administered anti-sense oligonucleotides (ASONs) targeting TGF-β to reverse the immunosuppressive environment of mesothelioma. We showed that intratumoral delivery of these agents resulted in the accumulation of T cells in the tumor and the slowing of mesothelioma tumor growth (Fitzpatrick et al., 1994; Marzo et al., 1997). Intratumoral delivery of the cytokine interleukin-2, had little effect by itself (Jackaman et al., 2003) but when combined with anti-CD40, caused local regression and, importantly regression of a distal untreated tumor (Jackaman et al., 2012), possibly due to the induction of inflammation within the tumor (Jackaman et al., 2008). Similar results were observed when agents such as toll-like receptor (TLR) seven agonists were delivered intratumorally—they had little affect alone but were synergistic with anti-CD40 (Currie et al., 2008; Broomfield et al., 2009). These novel loco-regional therapies provide a foundation for novel approaches to mesothelioma because of the accessibility of the tumor and are summarized in (Nelson et al., 2006) and (Nelson et al., 2014).

One of our earliest observations was that the adoptive transfer of large numbers of mesothelioma-specific T cells, CD4 and/or CD8, did not result in eradication of an established tumor (Marzo et al., 1999a), raising concerns for us that any such therapy alone, such as modern CAR-T-cell therapy, may not be effective in solid tumors unless the obstacles of effector site resistance to immune attack could be overcome, something being studied in a number of centers.

#### 3.4.1 Clinical Trials and Translation

These studies led directly to a clinical trial in mesothelioma conducted using local cytokine administration intratumorally, delivered continuously for 8 weeks (to mimic an inflammatory/immune disease process) via a catheter that was inserted under CT guidance. The recombinant cytokines granulocyte-macrophage colony-stimulating factor (GM-CSF) or IFNα were delivered continuously by a pump worn by the patient, with multiple side holes in the catheter ensuring that the cytokines were delivered to a large volume of tumor around the catheter. In a pilot study using IFNα we showed that such an approach was feasible, and two of the six patients showed responses, one with a generalized immune infiltrate and a diffuse partial response (Davidson et al., 1998). We studied 14 mesothelioma patients in this way using infused recombinant human GM-CSF. Although two patients responded to this therapy, however, neutrophil plugging of blood vessels occurred at some doses. Overall, this study demonstrated that intralesional infusion of cytokines is feasible and can lead to localized immune reaction with tumor regression in a minority of patients. However, the procedure can be associated with systemic toxicity and was associated with considerable technical problems (Davidson et al., 1998; Robinson et al., 1998).

## 4 Combining Chemotherapy With Immunotherapy

**FIGURE 3:**
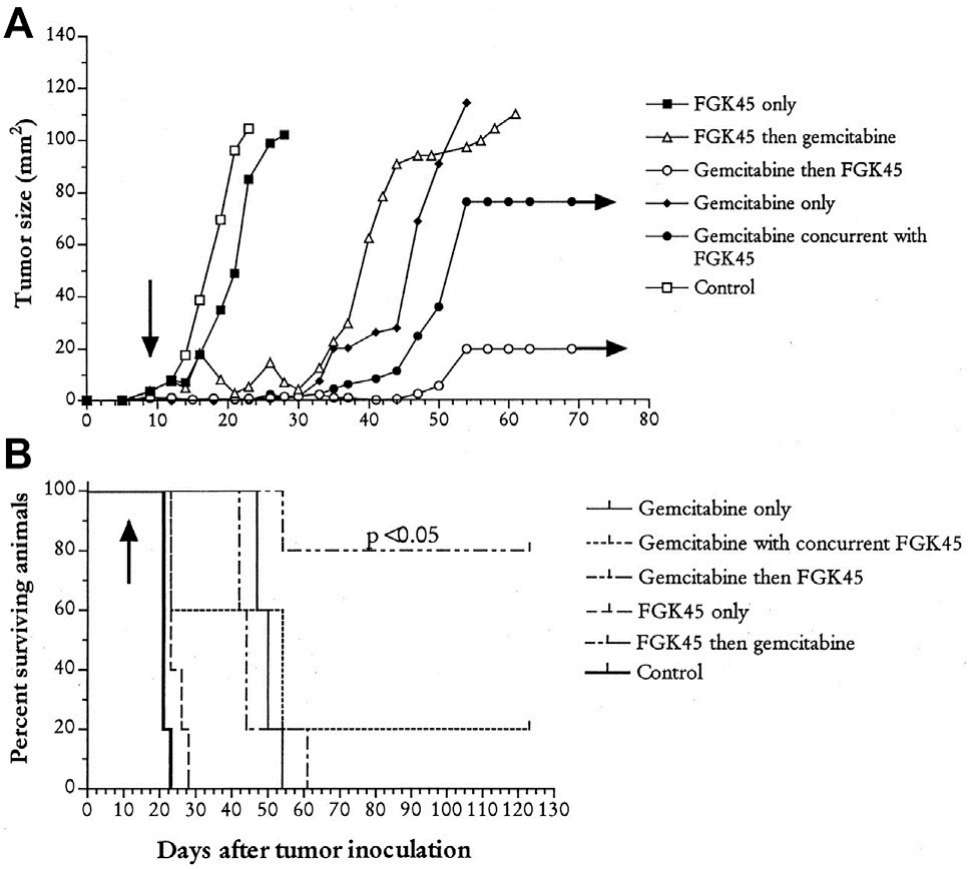
Synergy between chemotherapy and immunotherapy. Mice were injected with mesothelioma AB1-HA tumor cells and then treated on day nine with 120 ug/Gram gemcitabine intraperitoneally and/or 100 ug of FGK45 (activating anti-CD40) three times. Groups of mice received indicated treatment regimens; control animals were treated with PBS **(A)** Tumor growth rates and **(B)** survival curves. Arrows represent the start of treatment. Figure originally published in Nowak et al., 2003b. Journal of Immunology 170; 4905-4913.

Chemotherapy is immunosuppressive, and for this reason, was previously avoided in cancer immunotherapy trials. Using the murine mesothelioma model, we revisited this hypothesis and tested the effect of chemotherapy on neoantigen cross presentation (Nowak et al., 2003a) as well as the effect of combination chemotherapy with low dose anti-CD40 therapy. In both models, rather than observing a reduction in the anti-mesothelioma response, we demonstrated augmented responses, with mesothelioma cure in the majority of mice, even in mice with well-established tumors (Nowak et al., 2003a; Nowak et al., 2003b) (Figure 3). This seminal work then led to a number of studies evaluating synergy between chemotherapy and immunotherapy, showing that not all chemotherapy synergized with immunotherapy but that the phenomenon was robust and seen with different agents (van der Most et al., 2005; van der Most et al., 2006b). These results are summarized in (Lake and Robinson, 2005; Nowak et al., 2006).

This work also suggested that some/much of the anti-tumor effects of chemotherapy are mediated by the immune system as it responds to chemotherapy-induced cell death. Although our initial studies show that the immunotherapy agents anti-CTLA4 and anti-PD one had minimal effects in our mouse mesothelioma models as stand-alone treatments, we found combinations were effective (Fear et al., 2018). In addition, using network analysis, the team identified the biological features that underpinned effective anti-mesothelioma immune responses to checkpoint blockade (Lesterhuis et al., 2015).

### 4.1 Clinical Trials and Translation

Our first chemo-immunotherapy trial using Adriamycin and IFNα was disappointing, with the chemotherapy adding nothing to the immunotherapy except severe side effects (Upham et al., 1993). However, the results of subsequent preclinical experiments led directly to the establishment of a clinical trial using activating anti-CD40 with chemotherapy in patients with mesothelioma (Nowak et al., 2015). Anti-CD40 with cisplatin-pemetrexed chemotherapy was safe and tolerable (despite most patients experiencing cytokine release syndrome). Objective response rates were similar to chemotherapy alone however 20% of patients achieved long-term survival.

A subsequent trial, the DREAM trial, used the chemotherapies, cisplatin and pemetrexed plus an anti-PD-L1 antibody durvalumab and demonstrated promising activity and an acceptable safety profile as a platform for a current, on-going, phase III study (Nowak et al., 2020).

## 5 Surgery and Mesothelioma

Although surgery for mesothelioma is conducted in some specialized centers as a therapeutic procedure, usually as part of a multimodality approach, it is not used in all centers for patients with mesothelioma. Our mesothelioma animal models have provided an ideal opportunity to study the relationship between mesothelioma surgery and other treatments. Surgery in animal models allows for evaluation of each of the key components of cancer surgery in patients, including complete or partial resection of the primary tumor, removal of tumor dLN and evaluation of adjuvant therapy for recurrent local or metastatic disease after primary tumor resection. We initially mastered the complex surgical techniques required for complete tumor resection in small mice then determined the relationship between mesothelioma surgery and adjuvant immunotherapy. Surprisingly, partial tumor resection was more beneficial than complete resection in some situations as the former appears to provide a source of tumor antigens that promotes on-going immunity (Broomfield et al., 2005). Importantly for translation, we also showed that tumor debulking could be synergistic with immunotherapies delivered as immune-gene therapy (Mukherjee et al., 2001), via activating anti-CD40 (Khong et al., 2013; Khong et al., 2014) or by using neoantigen-based vaccination therapy (Fisher et al., 2014).

As we had previously shown that mesothelioma antigen presentation that leads to T-cell activation only occurs within the tumor dLN (Marzo et al., 1999b), we were concerned that resection of the dLNs which occurs frequently in cancer surgery, would hinder T-cell activation and limit the efficacy of post-surgical adjuvant immunotherapy. Using our models, we showed that this was not the case - resection of tumor dLN does not compromise adjuvant immunotherapy, at least using anti-CD40 or immune checkpoint blockade agents. Indeed, once the dLNs are removed, tumor antigen cross-presentation moves downstream to be undertaken by other lymph nodes, recruiting more lymph nodes into the cross-priming process.

**FIGURE 4:**
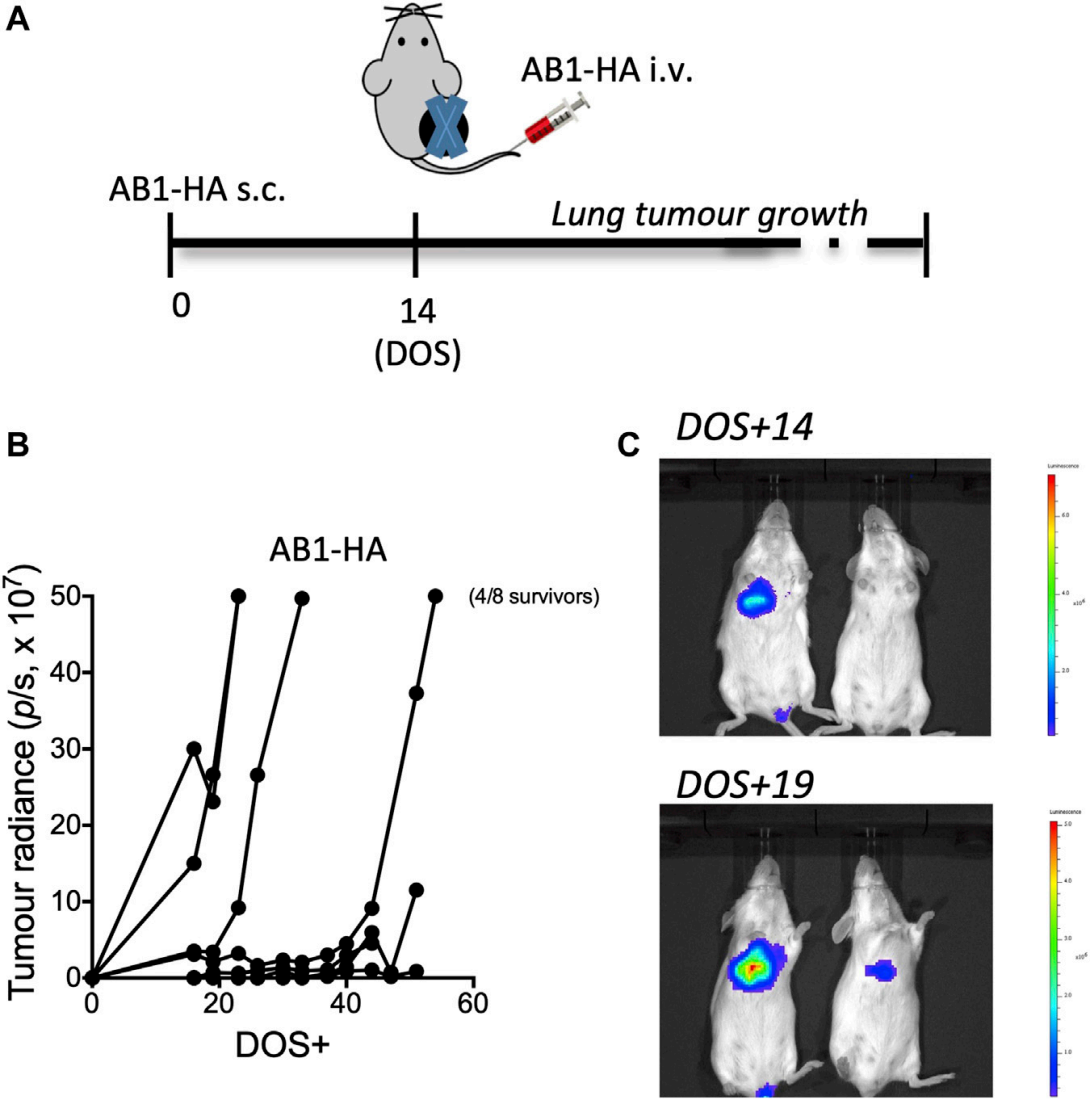
Early detection of metastatic lung disease by immune recognition versus PET-CT. To compare immune responses to neo-antigens with PET-CT in terms of sensitivity to recurrence of metastatic tumour. Mice received AB1-HA cells day 0 and AB1-HA_LUC cells intravenously on day 14. Tumours were surgically resected to mimic cancer surgery and lung tumour growth, as a measure of the appearance of metastases, was measured in an invitro imaging system (IVIS) **(A)** Experimental plan **(B)** lung tumour growth by IVIS **(C)** IVIS images of mice. DOS—day of surgery. Figure originally published in Fear et al., 2019. Scientific Reports 9:14,640.

We also showed that mesothelioma tumor growth could be tracked in live animals using *in vivo* imaging and PET-CT scanning. We demonstrated that tumor specific immune responses were very sensitive to the presence of post-surgical tumor recurrence, indicating the presence of tumor long before it was visible on PET-CT scanning (Fear et al., 2019) (Figure 4).

These studies have reduced concerns about the potential failure of adjuvant immunotherapy following dLN resection but further translation of these surgical findings into novel surgical studies, such as debulking surgery combined with adjuvant immunotherapy, is awaited.

## 6 Neoantigens and Mesothelioma Tumor Vaccines

**FIGURE 5:**
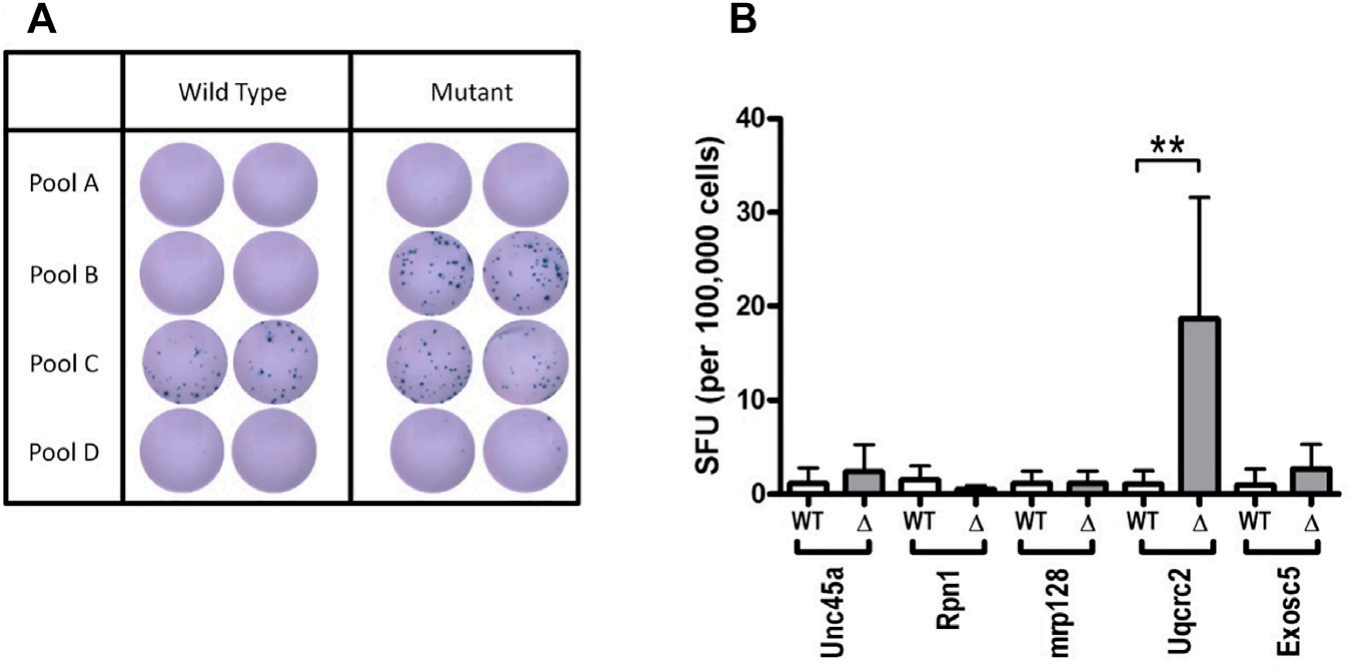
Immune responses to candidate asbestos-induced neo-antigens in murine mesothelioma. Immune responses against a panel of neo-antigen candidates predicted from genome sequencing **(A)** Representative duplicate wells from interferon-γ ELISPOT analysis of total tumour draining lymph node cell preparations from non-treated AB1 tumor-bearing mice against pools of predicted neo-antigenic peptides **(B)** Summary of ELISPOT data showing mean ± SD for the deconvolution of peptide Pool B. Mutant (*Δ*) peptide; wildtype (WT) peptide for indicated genes harboring single nucleotide variants. Figure originally published in Creaney et al., 2015. Oncoimmunology 4: e1011492.

Another strength of the murine mesothelioma models is the capacity to undertake detailed molecular analysis. We sequenced a large number of murine mesothelioma lines and identified the number and pattern of mutations in these tumors (Sneddon et al., 2017). Mutations can give rise to aberrant proteins that when recognized by the immune system are known as neoantigens (Ye et al., 2019). Using our genomics data we were able to predict and confirm the immunogenicity of several neoantigens (Creaney et al., 2015) (Figure 5) and these responses were useful in predicting the outcome immune check point inhibition therapy (Ma et al., 2020).

Ultimately the goal of neoantigen studies in our pre-clinical models is the production and validation of neoantigen vaccines. This is a novel and exciting new approach to tumor immunotherapy that has the capacity to augment immune checkpoint inhibitor therapy or function as a stand-alone treatment. To this end we are using the pre-clinical mouse mesothelioma models to validate our pipeline for the detection and validation of neoantigens, then using this information we will design novel approaches to the production and effective translation of neoantigen vaccines from mice to human.

Our neoantigen studies have led directly to a neoantigen clinical trial (ATTAC-001) in non-small cell lung cancer patients (Chee et al., 2017). We anticipate that the mouse studies will be run concurrently with future clinical trials in mesothelioma and other cancers in a true co-clinical fashion which will allow feedback from the bench-to-the-bedside and from the bedside-to-the-bench. Our studies showing that neoantigen T-cell responses are more sensitive to cancer recurrence than PET-CT imaging (Fear et al., 2019) could also pave the way for novel immunological approaches for monitoring tumor recurrence following conventional therapy, or novel immunotherapy approaches, including neoantigen vaccination.

## 7 Conclusion

In conclusion, our inbred mouse preclinical models of mesothelioma have represented a useful platform for translation of research findings into the clinic in the form of novel clinical trials. The current and future success of mesothelioma immunotherapy is partly founded on such models.

## References

[B1] Anyaegbu C. C. Lake R. A. Heel K. Robinson B. W. Fisher S. A. (2014). Chemotherapy Enhances Cross-Presentation of Nuclear Tumor Antigens. PLoS One 9 (9), e107894. 10.1371/journal.pone.0107894 25243472 PMC4171494

[B2] Aston W. J. Hope D. E. Nowak A. K. Robinson B. W. Lake R. A. Lesterhuis W. J. (2017). A Systematic Investigation of the Maximum Tolerated Dose of Cytotoxic Chemotherapy with and without Supportive Care in Mice. Bmc Cancer 17 (1), 684–710. 10.1186/s12885-017-3677-7 29037232 PMC5644108

[B3] Bielefeldt-Ohmann H. Fitzpatrick D. R. Marzo A. L. Jarnicki A. G. Himbeck R. P. Davis M. R. (1994). Patho- and Immunobiology of Malignant Mesothelioma: Characterisation of Tumour Infiltrating Leucocytes and Cytokine Production in a Murine Model. Cancer Immunol. Immunother. 39 (6), 347–359. 10.1007/BF01534421 8001022 PMC11041107

[B4] Bielefeldt-Ohmann H. Fitzpatrick D. R. Marzo A. L. Jarnicki A. G. Musk A. W. Robinson B. W. (1995). Potential for Interferon-Alpha-Based Therapy in Mesothelioma: Assessment in a Murine Model. J. Interferon Cytokine Res. 15 (3), 213–223. 10.1089/jir.1995.15.213 7584666

[B5] Broomfield S. Currie A. van der Most R. G. Brown M. van Bruggen I. Robinson B. W. (2005). Partial, but Not Complete, Tumor-Debulking Surgery Promotes Protective Antitumor Memory when Combined with Chemotherapy and Adjuvant Immunotherapy. Cancer Res. 65 (17), 7580–7584. 10.1158/0008-5472.CAN-05-0328 16140921

[B6] Broomfield S. A. Van Der Most R. G. Prosser A. C. Mahendran S. Tovey M. G. Smyth M. J. (2009). Locally Administered TLR7 Agonists Drive Systemic Antitumor Immune Responses that Are Enhanced by Anti-CD40 Immunotherapy. J. Immunol. 182 (9), 5217–5224. 10.4049/jimmunol.0803826 19380767

[B7] Byrne M. J. Davidson J. A. Musk A. W. Dewar J. van Hazel G. Buck M. (1999). Cisplatin and Gemcitabine Treatment for Malignant Mesothelioma: A Phase II Study. J. Clin. Oncol. 17 (1), 25–30. 10.1200/JCO.1999.17.1.25 10458214

[B8] Caminschi I. Venetsanakos E. Leong C. C. Garlepp M. J. Scott B. Robinson B. W. (1998). Interleukin-12 Induces an Effective Antitumor Response in Malignant Mesothelioma. Am. J. Respir. Cel Mol Biol 19 (5), 738–746. 10.1165/ajrcmb.19.5.3257m 9806738

[B9] Chee J. Robinson B. W. Holt R. A. Creaney J. (2017). Immunotherapy for Lung Malignancies: From Gene Sequencing to Novel Therapies. Chest 151 (4), 891–897. 10.1016/j.chest.2016.10.007 27769776

[B10] Christmas T. I. Manning L. S. Garlepp M. J. Musk A. W. Robinson B. W. (1993). Effect of Interferon-Alpha 2a on Malignant Mesothelioma. J. Interferon Res. 13 (1), 9–12. 10.1089/jir.1993.13.9 8454913

[B11] Creaney J. Ma S. Sneddon S. A. Tourigny M. R. Dick I. M. Leon J. S. (2015). Strong Spontaneous Tumor Neoantigen Responses Induced by a Natural Human Carcinogen. Oncoimmunology 4 (7), e1011492. 10.1080/2162402X.2015.1011492 26140232 PMC4485777

[B12] Currie A. J. van der Most R. G. Broomfield S. A. Prosser A. C. Tovey M. G. Robinson B. W. (2008). Targeting the Effector Site with IFN-Alphabeta-Inducing TLR Ligands Reactivates Tumor-Resident CD8 T Cell Responses to Eradicate Established Solid Tumors. J. Immunol. 180 (3), 1535–1544. 10.4049/jimmunol.180.3.1535 18209049

[B13] Davidson J. A. Musk A. W. Wood B. R. Morey S. Ilton M. Yu L. L. (1998). Intralesional Cytokine Therapy in Cancer: A Pilot Study of GM-CSF Infusion in Mesothelioma. J. Immunother. 21 (5), 389–398. 10.1097/00002371-199809000-00007 9789201

[B14] Davis M. R. Manning L. S. Whitaker D. Garlepp M. J. Robinson B. W. (1992). Establishment of a Murine Model of Malignant Mesothelioma. Int. J. Cancer 52 (6), 881–886. 10.1002/ijc.2910520609 1459729

[B15] Davis M. R. Manning L. S. Whitaker D. Garlepp M. J. Robinson B. W. S. (1993). A Murine Model of Mesothelioma. Eur. Respir. Rev. 3 (11), 116–117.

[B16] Fear V. S. Tilsed C. Chee J. Forbes C. A. Casey T. Solin J. N. (2018). Combination Immune Checkpoint Blockade as an Effective Therapy for Mesothelioma. Oncoimmunology 7 (10), e1494111. 10.1080/2162402X.2018.1494111 30288361 PMC6169578

[B17] Fear V. S. Forbes C. A. Chee J. Ma S. Neeve S. Celliers L. (2019). Neo-Antigen Specific T Cell Responses Indicate the Presence of Metastases before Imaging. Sci. Rep. 9 (1), 14640. 10.1038/s41598-019-51317-3 31601975 PMC6787183

[B18] Fisher S. A. Cleaver A. Lakhiani D. D. Khong A. Connor T. Wylie B. (2014). Neoadjuvant Anti-Tumor Vaccination Prior to Surgery Enhances Survival. J. Transl Med. 12 (1), 245–249. 10.1186/s12967-014-0245-7 25186961 PMC4156969

[B19] Fitzpatrick D. R. Bielefeldt-Ohmann H. Himbeck R. P. Jarnicki A. G. Marzo A. L. Robinson B. W. (1994). Transforming Growth Factor-Beta: Antisense RNA-Mediated Inhibition Affects Anchorage-Independent Growth, Tumorigenicity and Tumor-Infiltrating T-Cells in Malignant Mesothelioma. Growth Factors 11 (1), 29–44. 10.3109/08977199409015049 7833058

[B20] Fitzpatrick D. R. Manning L. S. Musk A. W. Robinson B. W. Bielefeldt-Ohmann H. (1995). Potential for Cytokine Therapy of Malignant Mesothelioma. Cancer Treat. Rev. 21 (3), 273–288. 10.1016/0305-7372(95)90004-7 7656268

[B21] Gibson P. G. Robinson B. W. McLennan G. Bryant D. H. Breit S. N. (1989). The Role of Bronchoalveolar Lavage in the Assessment of Diffuse Lung Diseases. Aust. N. Z. J. Med. 19 (3), 281–291. 10.1111/j.1445-5994.1989.tb00263.x 2775050

[B22] Jackaman C. Bundell C. S. Kinnear B. F. Smith A. M. Filion P. van Hagen D. (2003). IL-2 Intratumoral Immunotherapy Enhances CD8+ T Cells that Mediate Destruction of Tumor Cells and Tumor-Associated Vasculature: A Novel Mechanism for IL-2. J. Immunol. 171 (10), 5051–5063. 10.4049/jimmunol.171.10.5051 14607902

[B23] Jackaman C. Lew A. M. Zhan Y. Allan J. E. Koloska B. Graham P. T. (2008). Deliberately Provoking Local Inflammation Drives Tumors to Become Their Own Protective Vaccine Site. Int. Immunol. 20 (11), 1467–1479. 10.1093/intimm/dxn104 18824504

[B24] Jackaman C. Lansley S. Allan J. E. Robinson B. W. Nelson D. J. (2012). IL-2/CD40-Driven NK Cells Install and Maintain Potency in the Anti-mesothelioma Effector/Memory Phase. Int. Immunol. 24 (6), 357–368. 10.1093/intimm/dxs005 22354912

[B25] Khong A. Nelson D. J. Nowak A. K. Lake R. A. Robinson B. W. (2012). The Use of Agonistic Anti-CD40 Therapy in Treatments for Cancer. Int. Rev. Immunol. 31 (4), 246–266. 10.3109/08830185.2012.698338 22804570

[B26] Khong A. Brown M. D. Vivian J. B. Robinson B. W. Currie A. J. (2013). Agonistic Anti-CD40 Antibody Therapy Is Effective against Postoperative Cancer Recurrence and Metastasis in a Murine Tumor Model. J. Immunother. 36 (7), 365–372. 10.1097/CJI.0b013e31829fb856 23924788

[B27] Khong A. Cleaver A. L. Fahmi Alatas M. Wylie B. C. Connor T. Fisher S. A. (2014). The Efficacy of Tumor Debulking Surgery Is Improved by Adjuvant Immunotherapy Using Imiquimod and Anti-CD40. Bmc Cancer 14 (1), 969–9. 10.1186/1471-2407-14-969 25518732 PMC4320570

[B28] Krarup-Hansen A. Hansen H. H. (1991). Chemotherapy in Malignant Mesothelioma: A Review. Cancer Chemother. Pharmacol. 28 (5), 319–330. 10.1007/BF00685684 1914074

[B29] Kurts C. Robinson B. W. Knolle P. A. (2010). Cross-Priming in Health and Disease. Nat. Rev. Immunol. 10 (6), 403–414. 10.1038/nri2780 20498667

[B30] Lake R. A. Robinson B. W. (2005). Immunotherapy and Chemotherapy-Aa Practical Partnership. Nat. Rev. Cancer 5 (5), 397–405. 10.1038/nrc1613 15864281

[B31] Lee Y. C. Thompson R. I. Dean A. C. Robinson B. W. S. (2002). “Clinical and Palliative Care Aspects of Maligant Mesothelioma,” in Mesothelioma. Editors Robinson BWS Chahinian PA (London: Martin Dunitz), 111–126.

[B32] Leong C. C. Robinson B. W. Garlepp M. J. (1994). Generation of an Antitumour Immune Response to a Murine Mesothelioma Cell Line by the Transfection of Allogeneic MHC Genes. Int. J. Cancer 59 (2), 212–216. 10.1002/ijc.2910590213 7927922

[B33] Leong C. C. Marley J. V. Robinson B. W. Garlepp M. J. (1995). Antitumor Immune-Responses Generated by B7 Transfection in Murine Mesothelioma Cells of Disparate Immunogenicities. J. Celllular Biochem. 59, 174.

[B34] Leong C. Marley J. Loh S. Robinson B. Garlepp M. (1996). Induction and Maintenance of T-Cell Response to a Nonimmunogenic Murine Mesothelioma Cell Line Requires Expression of B7-1 and the Capacity to Upregulate Class II Major Histocompatibility Complex Expression. Cancer Gene Ther. 3 (5), 321–330. 8894251

[B35] Leong C. C. Marley J. V. Loh S. Robinson B. W. Garlepp M. J. (1997). The Induction of Immune Responses to Murine Malignant Mesothelioma by IL-2 Gene Transfer. Immunol. Cel Biol 75 (4), 356–359. 10.1038/icb.1997.55 9315477

[B36] Lesterhuis W. J. Rinaldi C. Jones A. Rozali E. N. Dick I. M. Khong A. (2015). Network Analysis of Immunotherapy-Induced Regressing Tumours Identifies Novel Synergistic Drug Combinations. Sci. Rep. 5, 12298. 10.1038/srep12298 26193793 PMC4508665

[B37] Lyons A. B. Parish C. R. (1994). Determination of Lymphocyte Division by Flow Cytometry. J. Immunol. Methods 171 (1), 131–137. 10.1016/0022-1759(94)90236-4 8176234

[B38] Ma S. Chee J. Fear V. S. Forbes C. A. Boon L. Dick I. M. (2020). Pre-Treatment Tumor Neo-Antigen Responses in Draining Lymph Nodes Are Infrequent but Predict Checkpoint Blockade Therapy Outcome. Oncoimmunology 9 (1), 1684714. 10.1080/2162402X.2019.1684714 32002299 PMC6959436

[B39] Manning L. S. Davis M. R. Bielefeldtohmann H. Marzo A. L. Garlepp M. J. Whitaker D. (1993). Evaluation of Immunogenicity of Murine Mesothelioma Cells by Immunization. Eur. Respir. Rev. 3 (11), 234–237.

[B40] Marzo A. L. Fitzpatrick D. R. Robinson B. W. Scott B. (1997). Antisense Oligonucleotides Specific for Transforming Growth Factor Beta2 Inhibit the Growth of Malignant Mesothelioma Both *In Vitro* and *In Vivo* . Cancer Res. 57 (15), 3200–3207. 9242450

[B41] Marzo A. L. Lake R. A. Robinson B. W. Scott B. (1999). T-cell Receptor Transgenic Analysis of Tumor-specific CD8 and CD4 Responses in the Eradication of Solid Tumors. Cancer Res. 59 (5), 1071–1079. 10070965

[B42] Marzo A. L. Lake R. A. Lo D. Sherman L. McWilliam A. Nelson D. (1999). Tumor Antigens Are Constitutively Presented in the Draining Lymph Nodes. J. Immunol. 162 (10), 5838–5845. 10229818

[B43] Marzo A. L. Kinnear B. F. Lake R. A. Frelinger J. J. Collins E. J. Robinson B. W. (2000). Tumor-Specific CD4+ T Cells Have a Major "Post-Licensing" Role in CTL Mediated Anti-Tumor Immunity. J. Immunol. 165 (11), 6047–6055. 10.4049/jimmunol.165.11.6047 11086036

[B44] McDonnell A. M. Robinson B. W. Currie A. J. (2010). Tumor Antigen Cross-Presentation and the Dendritic Cell: Where it All Begins? Clin. Dev. Immunol. 2010, 539519. 10.1155/2010/539519 20976125 PMC2957101

[B45] McDonnell A. M. Lesterhuis W. J. Khong A. Nowak A. K. Lake R. A. Currie A. J. (2015). Tumor-Infiltrating Dendritic Cells Exhibit Defective Cross-Presentation of Tumor Antigens, but Is Reversed by Chemotherapy. Eur. J. Immunol. 45 (1), 49–59. 10.1002/eji.201444722 25316312

[B46] McDonnell A. M. Joost Lesterhuis W. Khong A. Nowak A. K. Lake R. A. Currie A. J. (2015). Restoration of Defective Cross-Presentation in Tumors by Gemcitabine. Oncoimmunology 4 (5), e1005501. 10.1080/2162402X.2015.1005501 26155402 PMC4485774

[B47] Mukherjee S. Haenel T. Himbeck R. Scott B. Ramshaw I. Lake R. A. (2000). Replication-Restricted Vaccinia as a Cytokine Gene Therapy Vector in Cancer: Persistent Transgene Expression Despite Antibody Generation. Cancer Gene Ther. 7 (5), 663–670. 10.1038/sj.cgt.7700133 10830713

[B48] Mukherjee S. Nelson D. Loh S. van Bruggen I. Palmer L. J. Leong C. (2001). The Immune Anti-tumor Effects of GM-CSF and B7-1 Gene Transfection are Enhanced by Surgical Debulking of Tumor. Cancer Gene Ther. 8 (8), 580–588. 10.1038/sj.cgt.7700347 11571536

[B49] Nelson D. J. Robinson B. W. Allan J. van der Most R. (2005). Gene Therapy of Mesothelioma. Expert Opin. Biol. Ther. 5 (8), 1039–1049. 10.1517/14712598.5.8.1039 16050782

[B50] Nelson D. Jackaman C. Robinson B. W. S. Zhan Y. Koloska B. Mladinovic A. (2006). Targeting the Tumor Microenvironment: A Novel Immunotherapy that Cures Large Mesothelioma Tumors by Engaging Neutrophils, CD8+ T Cells and Tumor Blood Vessels. Lung Cancer 54 (54), S12. 10.1016/s0169-5002(07)70123-x

[B51] Nelson D. Fisher S. Robinson B. (2014). The "Trojan Horse" Approach to Tumor Immunotherapy: Targeting the Tumor Microenvironment. J. Immunol. Res. 2014, 789069. 10.1155/2014/789069 24955376 PMC4052171

[B52] Nowak A. K. Robinson B. W. Lake R. A. (2002). Gemcitabine Exerts a Selective Effect on the Humoral Immune Response: Implications for Combination Chemo-Immunotherapy. Cancer Res. 62 (8), 2353–2358. 11956096

[B53] Nowak A. K. Byrne M. J. Williamson R. Ryan G. Segal A. Fielding D. (2002). A Multicentre Phase II Study of Cisplatin and Gemcitabine for Malignant Mesothelioma. Br. J. Cancer 87 (5), 491–496. 10.1038/sj.bjc.6600505 12189542 PMC2376155

[B54] Nowak A. K. Lake R. A. Kindler H. L. Robinson B. W. (2002). New Approaches for Mesothelioma: Biologics, Vaccines, Gene Therapy, and Other Novel Agents. Semin. Oncol. 29 (1), 82–96. 10.1053/sonc.2002.30234 11836673

[B55] Nowak A. K. Lake R. A. Marzo A. L. Scott B. Heath W. R. Collins E. J. (2003). Induction of Tumor Cell Apoptosis *In Vivo* Increases Tumor Antigen Cross-Presentation, Cross-Priming rather Than Cross-Tolerizing Host Tumor-Specific CD8 T Cells. J. Immunol. 170 (10), 4905–4913. 10.4049/jimmunol.170.10.4905 12734333

[B56] Nowak A. K. Robinson B. W. Lake R. A. (2003). Synergy between Chemotherapy and Immunotherapy in the Treatment of Established Murine Solid Tumors. Cancer Res. 63 (15), 4490–4496. 12907622

[B57] Nowak A. K. Byrne M. J. Millward M. J. Alvarez J. M. Robinson B. W. (2004). Current Chemotherapeutic Treatment of Malignant Pleural Mesothelioma. Expert Opin. Pharmacother. 5 (12), 2441–2449. 10.1517/14656566.5.12.2441 15571462

[B58] Nowak A. K. Lake R. A. Robinson B. W. (2006). Combined Chemoimmunotherapy of Solid Tumours: Improving Vaccines? Adv. Drug Deliv. Rev. 58 (8), 975–990. 10.1016/j.addr.2006.04.002 17005292

[B59] Nowak A. K. Cook A. M. McDonnell A. M. Millward M. J. Creaney J. Francis R. J. (2015). A Phase 1b Clinical Trial of the CD40-Activating Antibody CP-870,893 in Combination with Cisplatin and Pemetrexed in Malignant Pleural Mesothelioma. Ann. Oncol. 26 (12), 2483–2490. 10.1093/annonc/mdv387 26386124

[B60] Nowak A. K. Lesterhuis W. J. Kok P. S. Brown C. Hughes B. G. Karikios D. J. (2020). Durvalumab with First-Line Chemotherapy in Previously Untreated Malignant Pleural Mesothelioma (DREAM): A Multicentre, Single-Arm, Phase 2 Trial with a Safety Run-In. Lancet Oncol. 21 (9), 1213–1223. 10.1016/S1470-2045(20)30462-9 32888453

[B61] Robinson B. W. S. Manning L. S. Bowman R. V. Christmas T. I. Musk A. W. Davis M. R. (1993). The Scientific Basis for the Immunotherapy of Human-Malignant Mesothelioma. Eur. Respir. Rev. 3 (11), 195–198.

[B62] Robinson B. W. Mukherjee S. A. Davidson A. Morey S. Musk A. W. Ramshaw I. (1998). Cytokine Gene Therapy or Infusion as Treatment for Solid Human Cancer. J. Immunother. 21 (3), 211–217. 10.1097/00002371-199805000-00007 9610913

[B63] Robinson B. W. Lake R. A. Nelson D. J. Scott B. A. Marzo A. L. (1999). Cross-Presentation of Tumour Antigens: Evaluation of Threshold, Duration, Distribution and Regulation. Immunol. Cel Biol 77 (6), 552–558. 10.1046/j.1440-1711.1999.00876.x 10571677

[B64] Robinson B. W. Robinson C. Lake R. A. (2001). Localised Spontaneous Regression in Mesothelioma -- Possible Immunological Mechanism. Lung Cancer 32 (2), 197–201. 10.1016/s0169-5002(00)00217-8 11325491

[B65] Sneddon S. Patch A. M. Dick I. M. Kazakoff S. Pearson J. V. Waddell N. (2017). Whole Exome Sequencing of an Asbestos-Induced Wild-Type Murine Model of Malignant Mesothelioma. BMC cancer 17 (1), 396. 10.1186/s12885-017-3382-6 28577549 PMC5455120

[B66] Sterman D. H. Haas A. Moon E. Recio A. Schwed D. Vachani A. (2011). A Trial of Intrapleural Adenoviral-Mediated Interferon-α2b Gene Transfer for Malignant Pleural Mesothelioma. Am. J. Respir. Crit. Care Med. 184 (12), 1395–1399. 10.1164/rccm.201103-0554CR 21642245 PMC3262033

[B67] Stumbles P. A. Himbeck R. Frelinger J. A. Collins E. J. Lake R. A. Robinson B. W. (2004). Cutting Edge: Tumor-specific CTL Are Constitutively Cross-Armed in Draining Lymph Nodes and Transiently Disseminate to Mediate Tumor Regression Following Systemic CD40 Activation. J. Immunol. 173 (10), 5923–5928. 10.4049/jimmunol.173.10.5923 15528325

[B68] Upham J. W. Musk A. W. van Hazel G. Byrne M. Robinson B. W. (1993). Interferon Alpha and Doxorubicin in Malignant Mesothelioma: A Phase II Study. Aust. N. Z. J. Med. 23 (6), 683–687. 10.1111/j.1445-5994.1993.tb04727.x 8141698

[B69] Upham J. W. Holt B. J. Baron-Hay M. J. Yabuhara A. Hales B. J. Thomas W. R. (1995). Inhalant Allergen-Specific T-Cell Reactivity Is Detectable in Close to 100% of Atopic and Normal Individuals: Covert Responses are Unmasked by Serum-Free Medium. Clin. Exp. Allergy 25 (7), 634–642. 10.1111/j.1365-2222.1995.tb01111.x 8521182

[B70] van der Most R. G. Robinson B. W. Lake R. A. (2005). Combining Immunotherapy with Chemotherapy to Treat Cancer. Discov. Med. 5 (27), 265–270. 20704886

[B71] van der Most R. G. Robinson B. W. Nelson D. J. (2006). Gene Therapy for Malignant Mesothelioma: Beyond the Infant Years. Cancer Gene Ther. 13 (10), 897–904. 10.1038/sj.cgt.7700935 16439992

[B72] van der Most R. G. Currie A. Robinson B. W. Lake R. A. (2006). Cranking the Immunologic Engine with Chemotherapy: Using Context to Drive Tumor Antigen Cross-Presentation towards Useful Antitumor Immunity. Cancer Res. 66 (2), 601–604. 10.1158/0008-5472.CAN-05-2967 16423984

[B73] van der Most R. G. Currie A. J. Mahendran S. Prosser A. Darabi A. Robinson B. W. (2009). Tumor Eradication after Cyclophosphamide Depends on Concurrent Depletion of Regulatory T Cells: A Role for Cycling TNFR2-Expressing Effector-Suppressor T Cells in Limiting Effective Chemotherapy. Cancer Immunol. Immunother. 58 (8), 1219–1228. 10.1007/s00262-008-0628-9 19052741 PMC11030690

[B74] Wagner J. C. Berry G. (1969). Mesotheliomas in Rats Following Inoculation with Asbestos. Br. J. Cancer 23 (3), 567–581. 10.1038/bjc.1969.70 5360333 PMC2008422

[B75] Ye L. Ma S. Robinson B. W. Creaney J. (2019). Immunotherapy Strategies for Mesothelioma - The Role of Tumor Specific Neoantigens in a new era of Precision Medicine. Expert Rev. Respir. Med. 13 (2), 181–192. 10.1080/17476348.2019.1563488 30596292

